# CBE and the Missing ‘E’: Making Competency-Based Education an Equitable Framework for Global Health Professions Education

**DOI:** 10.5334/pme.2551

**Published:** 2026-09-29

**Authors:** Jamiu O. Busari, Ming-Ka Chan, Arvin Damodaran, Alan Schwartz, Roberta Ladenheim, Ben Jones, Marjan Khajehei, David A. Turner, Liknaw Zeleke, Holly A. Caretta-Weyer, Members of the International Competency-Based Health Professions Educators Collaborative

**Affiliations:** 1 Faculty of Health, Medicine, and Life Sciences, Maastricht University, The Netherlands; 2 Horacio Oduber Hospital, Aruba; 3 Max Rady College of Medicine, Rady Faculty of Health Sciences, University of Manitoba, Canada; 4 School of Clinical Medicine, Faculty of Medicine and Health, UNSW Sydney, Australia; 5 University of Illinois Chicago, United States; 6 Universidad Hospital Italiano de Buenos Aires, Buenos Aires, Argentina; 7 Nuffield Department of Medicine, University of Oxford, Oxford, United Kingdom; 8 Menzies School of Health Research, Darwin, Australia; 9 Westmead Hospital, WSLHD, Australia; 10 University of Sydney, Australia; 11 University of New South Wales, Australia; 12 Innovation and Competency-Based Education, American Board of Pediatrics, Chapel Hill, NC, United States; 13 College of Health Sciences, Debre Markos University, Debre Markos, Ethiopia; 14 School of Women’s and Children’s Health, Faculty of Medicine, University of New South Wales, Sydney, Australia; 15 Stanford University School of Medicine in Palo Alto, California, United States

## Abstract

It is generally assumed that competency-based health professions education (CBHPE) is a positive and ongoing educational reform. However, the assumption that CBHPE naturally leads to more equitable outcomes has not been thoroughly examined. This ‘Eye Opener’ identifies global challenges in implementing equitable CBHPE, with particular attention to majority-world (low- and middle-income) contexts. These tensions include: 1) variable approaches to adoption that limit transferability, 2) uneven infrastructure for implementation and innovation, 3) export of predeveloped conceptual frameworks that may not be locally fit for purpose or implemented alongside collaborative knowledge exchange, and 4) pressures for fidelity in implementation that impede flexibility and compromise effectiveness in local contexts. In each area of tension, the authors outline challenges to equity and suggest potential ways forward. From their analysis, the authors propose that equity must be deliberately embedded in CBHPE at multiple levels, and the proposed solutions include addressing equity on three levels – the micro level of individual learner access and assessment; the meso level of curricula, faculty development, and program learning culture; and the macro level of national accreditation standards and education policies. Examples of opportunities at each level and for each of the five core components of CBHPE are also provided. The authors argue for a shift in perspective—from “outside-in,” in which Western nations export CBHPE to the majority world, to “outside-in, inside-out and all around,” in which CBHPE frameworks are co-created to leverage contributions from both Western and majority-world education systems. They believe that more thoughtful adaptation and embracing Global South ways of knowing, doing, and being, rather than simple replication, alongside the continuous application of a social justice lens, are needed to ensure that CBHPE serves all communities and contributes to equitable, high-quality learning and healthcare without discrimination.

## Introduction

### Finding the missing E

There is a general perception that competency-based health professions education (CBHPE) is a positive and ongoing educational reform. At the same time, CBHPE has also attracted critique, including concerns regarding reductionism, over-standardization, and fragmentation of professional practice. Unfortunately, less attention has been paid to how inequities may be reproduced through CBHPE implementation itself, particularly in majority-world (low- and middle-income) contexts, where there is growing concern about CBHPE’s capacity to produce equitable outcomes. In this ‘Eye Opener,’ we challenge the assumption that CBHPE is inherently progressive; instead, we interrogate whether CBHPE outcomes could be made more equitable. Given CBHPE’s ultimate aim to benefit society, attention to equity is essential to ensure that learners are representative of the patients and communities they serve and to achieve the outcomes required to provide the highest-quality care possible. We posit equity as the missing “E” in CBHPE. In the past decade, equity work has increasingly become embedded in healthcare and healthcare education. For example, equity initiatives have been associated with increased workforce diversity and have been shown to promote more inclusive and equitable healthcare cultures [1]. Furthermore, research shows that patients do better when cared for by a diverse healthcare team, i.e., a team that is better at managing change, communicating effectively, finding solutions, and dealing with risk [2]. In this paper, equity refers to fairness, inclusion, and justice in access to educational opportunities, learning environments, assessment practices, and professional progression within CBHPE systems [3].

Five core components define CBHPE: defined outcomes and competencies, a sequenced progression of competence, tailored learning experiences, competency-focused instruction, and a programmatic assessment approach [4]. Yet in practice, there is substantial heterogeneity in how CBHPE is implemented, evaluated, and reported worldwide. A recent review of competency-based postgraduate medical education (PGME) programs found wide variation in evaluation objectives, tools, and reporting standards [5]. Many programs failed to clarify evaluation designs or benchmarks, limiting cross-context learning and transferability. Despite recent (albeit controversial) calls for CBHPE frameworks to explicitly indicate equity components [6,7,8], we have identified very few that have done so [9,10], and other authors have reached similar conclusions within individual specialties [11].

With the ‘missing E’ in mind, the starting point for our globally representative author group (author reflexivity statement follows the article) was the presumption that thoughtful, contextually responsive adaptation, rather than blind replication, is essential if CBHPE is to serve health goals fairly and equitably across all jurisdictions in health professions education. Drawing on the lived experience and expertise of the assembled authors, as well as a purposive sampling of academic, government, and institutional publications, we highlight major contemporary topics in the CBHPE global equity landscape and propose potential solutions at the micro-, meso-, and macro-levels. Of note, this paper focuses on global North-South inequities and their impact on CBE implementation, with less attention to other forms of inequity linked to gender, religion, ethnicity, or ableness, for example.

## Adoption and transferability

### Challenges

In a globally connected world, health professional educators have an obligation to address critical issues of equitable adaptation, accessibility, and power dynamics in the importation and implementation of CBHPE in local contexts. CBHPE frameworks conceptualized in the global North are often the most well-known globally, and their validity, equity, and feasibility across *majority-world* (that is, low- and middle-income) contexts remain unclear [12,13]. Inclusive models advocate for learner-designed assessments and culturally relevant tools that acknowledge diverse experiences and promote justice-oriented competencies [3].

Health professions education literature consistently highlights the tension between CBHPE’s conceptual appeal and the practical realities of implementation. While CBHPE has undergone significant change, countries in Asia, the Middle East, Africa, the Caribbean, and Latin America have adopted CBHPE in varying ways, often integrating it through pilot programs, discipline-specific initiatives, or postgraduate reforms [14,15,16,17,18]. However, there are clear contextual departures in implementation required to respond to the adoption of CBHPE, particularly the use of imported frameworks in contexts that center on wholly different priorities in the provision of education and patient care [10,19]. Regardless of global region (e.g., North/South) and local income level, there remains a gap in the explicit attention to equity in access to curricular reform innovations, educational resources, and opportunities. Even in high-income regions with structured CBHPE frameworks, national accreditation standards, and digital tracking platforms, it does not appear that authentic and equitable implementation of these frameworks has been fully achieved [12,13].

### Proposed Solutions

Addressing this persistent gap requires repositioning equity as an explicit design principle within CBHPE rather than an assumed outcome of competency frameworks. This repositioning involves embedding equity expectations into competency descriptors, EPAs, and assessment rubrics, and strengthening institutional capacity to enact policy in ways that reduce rather than reproduce inequities. Practical actions include supporting educators and clinical supervisors to recognize how everyday decisions shape learner access, assessment fairness, and psychological safety; exploring and mapping structural barriers across placement allocation, supervision quality, digital access, and language load; and co-designing targeted interventions with learners and clinical partners. Building educator capability for culturally safe, bias-aware teaching, assessment, and summative decision-making, alongside moderated and narrative approaches, can further mitigate inequitable decisions [20]. Digital tracking systems must also be audited for accessibility and unintended bias, with alternatives provided for learners and faculty with limited digital or linguistic resources. Finally, embedding learner voices (especially those with one or more structurally excluded identities) in governance and monitoring equity outcomes, not only competency attainment, ensures that inequities are identified early and addressed through continuous, accountable improvement.

The common thread in CBHPE is the shift away from time-based progression toward demonstrable competence informed by programmatic assessment [21]. In the context of equitable CBHPE, this would mean designing programmatic assessments to be more authentic, culturally relevant, and equity-oriented. A well-documented example is the Royal College of Physicians and Surgeons of Canada’s Competence by Design reform, in which trainees progress by demonstrating competence in Entrustable Professional Activities (EPAs) supported by aggregated workplace-based assessments rather than fixed rotations [22]. Similarly, Dutch postgraduate programs have embedded EPAs within broader competency-based reforms, shifting advancement toward entrustment decisions grounded in observed performance [23,24]. In majority-world contexts, hands-on, learner-driven assessment plans, longitudinal coaching, and narrative-based evaluation can be of significant value and are increasingly recommended to address the limitations of standardized tools while acknowledging the diverse experiences learners bring to training [25,26]. Programmatic assessment models developed at Maastricht University, for example, illustrate this redesign, using longitudinal portfolios that integrate low-stakes assessments, narrative feedback, and multisource input [27]. Narrative feedback is prioritized over numeric scoring, and coaching supports learner development over time. Comparable systems in U.S. schools, including the Cleveland Clinic Lerner College of Medicine, also emphasize coaching, narrative evaluation, and developmental progression over traditional grading [28,29,30].

In summary, we do acknowledge that the examples described above are quite aspirational and may be daunting in some geographical/academic contexts. Nonetheless, we encourage innovators to share solutions based on their local contexts.

## Infrastructure and innovation

### Challenges

In majority-world countries, the institutional conditions for CBHPE are uneven, often due to resource constraints or contextual factors related to learner-faculty interactions and how systems function within these contexts to provide patient care under a variety of stressors and external pressures. For example, a recent study on competency-based PGME in Pakistan found that weak feedback systems, limited documentation of workplace-based assessment (WBA), lack of longitudinal continuity in clinical postings, and misalignment between institutional policies and resources hindered implementation [5]. That is not to say these issues are absent in high-income countries; some challenges lead to inconsistent implementation of CBHPE even in circumstances where resources are more abundant [31].

In addition to these challenges, programs within majority world countries and other resource-constrained settings are more likely to face a range of equity-related barriers in implementing CBHPE [5,32]. These include inadequate infrastructure, such as outdated facilities, limited access to simulation labs, poor internet connectivity, and lack of access to digital tools like electronic portfolios – all of which hinder the delivery of modern, competency-aligned curricula, feedback, and assessment. Faculty shortages and limited professional development opportunities reduce the capacity to effectively deliver and assess competencies, especially in clinical settings. Language barriers and cultural mismatches between imported competency frameworks and local healthcare realities can alienate learners and/or faculty and reduce relevance. Additionally, socioeconomic disparities among learners, such as rural-urban divides, gender inequities, and financial constraints, limit access to clinical placements, mentorship, and academic opportunities and support. These barriers require context-sensitive, locally driven solutions for the individual, groups, and programs to ensure that CBHPE fulfills its promise of equitable, high-quality education for all.

### Proposed Solutions

Equity is fundamentally a matter of educational planning and design. Although not every institution will have access to advanced technology or extensive resources, all programs can pursue CBHPE through an equity lens by implementing solutions such as coordinated supervision across sites, low-cost digital portfolios, and protected time for feedback within clinical workloads. In majority-world and other resource-constrained settings, uneven institutional conditions, driven by resource limitations, workforce pressures, and hierarchical learner–faculty dynamics, make equitable CBHPE difficult to implement without intentionality in design. Strengthening equity requires investment in basic teaching infrastructure, building faculty capability for fair and culturally aligned supervision and assessment, and co-designing CBHPE processes with local educators and learners. Transparent governance, supportive workload policies, and mechanisms to address inequitable treatment help stabilize learning environments under chronic stress. Monitoring access to clinical and other academic opportunities, supervision quality, and assessment outcomes enables institutions to identify where contextual pressures disadvantage particular learners or learner groups and to implement targeted, feasible improvements [33].

Although CBHPE implementation in resource-constrained environments is shaped by factors such as limited faculty capacity, inconsistent clinical/academic exposure, and infrastructure gaps, these settings can also demonstrate some of the most innovative adaptations. For example, majority-world programs may rely on community-based training, low-cost simulation, and competency portfolios to ensure learners achieve core outcomes despite material constraints. Decentralized, community-engaged training models—partnering with district hospitals and primary care clinics—expand authentic clinical exposure while aligning competencies with the needs of the local population [22,34]. The emphasis on equity is particularly salient: learners frequently serve populations facing structural disadvantage, making competencies in advocacy, cultural humility, and systems awareness essential [35,36,37]. Therefore, integrating competencies in advocacy, cultural humility, and health systems improvement can ensure alignment with the needs of structurally disadvantaged populations [38]. Literature from these contexts underscores that CBHPE can be a mechanism for strengthening health systems, provided that competencies are contextualized, assessments are feasible, and faculty development is prioritized [35,39]. Faculty capacity can be strengthened, for example, through train-the-trainer approaches, regional collaboratives, and virtual communities of practice that support feedback literacy and entrustment decision-making [23]. Flexible, scalable models are therefore central to ensuring that CBHPE does not exacerbate inequities between well-resourced and less-resourced programs or institutions [6]. Such flexible, contextually responsive models help prevent CBHPE from reproducing inequities while contributing to health system strengthening [28,40].

## Export versus exchange

### Challenges

Although CBHPE is often portrayed as objective and fair, its dominant frameworks, assessment logics, and entrustment processes are grounded in Western biomedical norms, reflecting specific cultural and institutional assumptions [39,40,41,42]. As a result, many majority-world institutions experience pressure to conform to these external standards, which in turn can facilitate graduate mobility to Western countries [43]. Hence, driven by ambition and economic realities, some institutions (especially in post-colonial countries) train medical graduates to satisfy the colonial standards [43,44]. For example, medical curricula in Anglophone countries such as Nigeria and Ghana may be structured to facilitate doctors’ transition to the UK and other Commonwealth countries, including Canada and Australia. In these cases, even though the degrees are recognized by the General Medical Council (GMC) in the UK, trainees still have to pass licensing exams, such as the Professional and Linguistic Assessments Board, and demonstrate competence to practice before they can work. Unfortunately, these standards frequently fail to account for local disease burdens, language differences, resource constraints, and cultural paradigms of the trainees from majority-world countries [45]. Furthermore, learners from structurally marginalized backgrounds often bear a disproportionate burden in adapting to imported norms, sometimes at the cost of their personal identity or community relevance [39,46].

In many contexts, initiatives to increase representation of Indigenous and other minoritized learners risk reproducing colonial hierarchies when implemented within essentially unchanged CBHPE structures [33,47,48]. Efforts that focus primarily on access or numbers may inadvertently position minoritized learners as needing to assimilate to pre-existing definitions of competence rather than questioning whose knowledge and practices those definitions privilege [49]. From a decolonial and anti-oppressive perspective, this constitutes and perpetuates epistemic injustice, as particular ways of knowing, caring, and practicing medicine are systematically undervalued [42,50]. Without deliberate attention to redefining competence and assessment, representation-focused reforms risk functioning as symbolic inclusion, increasing diversity without disrupting entrenched inequities in specialty training [33,47].

### Proposed Solutions

To counter the inequities created when CBHPE frameworks are imported from Western biomedical traditions, majority-world institutions need to adapt competency standards so they reflect local health priorities, cultural norms, and resource realities. This adaptation includes revising competencies and entrustment processes to account for local disease burdens, linguistic diversity, and relational models of care, and co-developing these frameworks with local educators, regulators, students, and communities. Developing and improving national accreditation systems can reduce pressure to conform to Western standards which favor graduate mobility over service to local communities, while faculty development focused on culturally grounded, context-sensitive assessment supports fairer implementation. Ongoing equity audits and feedback from learners and graduates ensure that CBHPE evolves in ways that serve local needs rather than reproducing external expectations.

An equitable CBHPE system should accommodate cross-border migration of learners and practitioners, ensuring they are competent in their training settings while enabling further development in new contexts. A corollary is that curricula should not be exported wholesale from setting to setting; instead, CBHPE should be equitable and promote the exchange of valuable practices, bidirectional knowledge flows, and contextual adaptations. For example, the East, Central, and Southern Africa Health Community adapted a South African competency-based nursing curriculum for Tanzania, Kenya, and Uganda, tailoring competencies, clinical examples, and faculty development to local health priorities while sharing lessons across countries [51]. Similarly, the Canadian Competence by Design frameworks have been adapted in Middle Eastern residency programs, aligning Entrustable Professional Activities with local patient populations and cultural norms through co-led delivery and virtual mentorship, enabling both adoption and local relevance [52]. Such contextually responsive approaches support global learner mobility without reproducing inequities.

## Flexibility versus fidelity

### Challenges

Many debates about CBHPE hinge on *fidelity* versus *flexibility* of CBHPE implementation in various educational contexts [4,53]. For example, the common practice of rigid insistence on “gold-standard” designs imported from well-resourced contexts risks perpetuating educational inequity in many educational programs. Those programs that cannot meet all design elements may be deemed inferior or noncompliant, thereby perpetuating existing hierarchies and stifling innovation. The battle between fidelity of implementation and flexibility is one of the great debates, and there are ongoing global challenges with fidelity of implementation—even in the most resource-rich settings. And in majority-world settings, slavish devotion to exported and retrofitted CBHPE approaches has the potential to exacerbate existing inequities.

Several underlying forces resist flexibility, resulting in frameworks that are misaligned with local needs and contexts [40,54]:

- **Preservation of power and control:** Interested parties invested in established assessment and accreditation models may view deviation from standardized CBHPE frameworks as a threat to professional credibility, legitimacy, or comparability, reinforcing existing hierarchies and epistemic dominance within HPE [42,55]. Accrediting bodies and regulatory agencies may resist ceding authority to local programs/institutions due to concerns about variability, accountability, and erosion of standards, leading to rigid implementation approaches that limit contextual equity [41,48].
- **Perceived supremacy of standardization:** There is often a presumption that strict uniformity ensures quality and fairness in CBHPE; however, over-reliance on standardized competencies and assessments may obscure contextual realities and exclude locally adaptive or culturally responsive models of competence [28,56]. Realist inquiry reminds us that context is imperative to consider. Focusing on what works, for whom, in what context, and why, significantly impacts successful implementation and captures meaningful contextual flexibilities necessary for local adaptation [57].
- **Limited worldview or epistemic insularity:** CBPHE decision-makers may operate within narrow conceptual paradigms that privilege dominant biomedical and Western epistemologies, undervaluing local knowledge systems, community-defined competencies, and alternative ways of demonstrating competence [42,49].
- **Policy fragmentation and system inertia:** Lack of coordination among ministries of health, educational/health institutions, and regulatory bodies, combined with institutional inertia, slow CBHPE reform and perpetuate inequitable implementation across regions and programs [40,48].

At the organizational level, resource constraints make fidelity particularly challenging, and necessitate local solutions, even as the resources for adaptation are themselves in short supply. Insufficient numbers of trained faculty and inconsistent access to faculty development in assessment, feedback, and coaching undermine CBHPE effectiveness [41,58]. Faculty development to support frontline educators in addressing bias and inequities that affect learners is equally critical yet inconsistently available. Inadequate access to educational technologies such as learning management systems, simulation centers, digital portfolios, and data-tracking tools limits learner engagement and the longitudinal assessment essential to the successful implementation of CBHPE [57,59,60]. Although these issues may be more obvious in the majority world, they often apply in resource-rich settings as well.

Finally, learners who would benefit most from flexibility are often those who are least able to resist pressures for fidelity. Financial constraints, rural–urban divides, gender inequities, and social marginalization shape learners’ access to clinical placements, mentorship, academic experiences, and support, resulting in unequal opportunities to demonstrate competence that might belie the need for an exact replication of models developed with well-resourced learners in mind [49,61]. The previously mentioned paucity of faculty and limited access to faculty development reduce opportunities for relationship-building among faculty and between faculty and learners.

### Proposed Solutions

Fidelity to practices shown to be effective in one setting becomes a hollow ideal when those practices are not fit for purpose elsewhere. CBHPE systems must adopt flexible approaches that serve common goals of social accountability while respecting local context. For example, a scoping review on competency-based education and training for community health workers (CHWs) showed that learning programs tailored to consider low literacy, content designed for local cultural contexts, and curricula co-created with CHWs were effective strategies for translating learning into practice [62]. Similarly, a distributed medical training program in South Africa emphasized co-creation of curricula and longitudinal coaching between learners and faculty across multiple district hospitals, allowing adaptation to variable clinical exposure and resource constraints [63]. These strategies illustrate that flexibility, local relationship-building, and experimentation support socially accountable CBHPE and strengthen learner competence while respecting and leveraging contextual realities.

To move beyond the fidelity versus flexibility debate in CBHPE, equity-focused implementation requires recognizing that strict adherence to imported models can marginalize learners. In contrast, unstructured flexibility can dilute standards and create inconsistency. A more productive approach is to adopt *principled adaptability*: maintaining the core intent of competencies – which includes safe, effective, and context-relevant practice – while allowing institutions to tailor assessment processes, supervision structures, and learning activities to local realities. For example, providing an immediate, tailored, and context- and resource-sensitive learning plan before a learner reattempts an equivalent assessment reflects principled adaptability.

This adaptation includes defining which elements of CBHPE must remain stable for fairness and comparability, and which can be adapted to reflect cultural norms, resource constraints, and local health priorities. Supporting educators to make transparent, well-reasoned adaptations and embedding mechanisms for shared decision-making and moderation ensures that flexibility enhances rather than undermines equity, educational quality, and the ultimate care of patients.

## A framework for equity

The tension between standardized competence and variable contexts becomes more pronounced when models developed in well-resourced settings are transplanted into under-resourced or otherwise divergent environments with varying supervisory capacity, clinical complexity, population burdens, or cultural norms. Operationalizing this framework across the micro (individual and interpersonal), meso (programmatic and team-based), and macro (institutional to global) spheres requires not only explicitly naming equity but also embedding it in curricular design, implementation, and evaluation.

While addressing all forms of inequity is clearly beyond the scope of this paper, we propose a multilevel framework for equity in competency-based assessment that addresses some of the disparities that may be seen between high- and low-income settings. Grounded in recent literature, Table 1 emphasizes how learner performance is shaped by context at the micro- (learner–supervisor–patient), meso- (program design and culture), and macro-levels (institutional and societal norms) [64]. The framework and the proposed solutions highlight how institutional and cultural contexts shape assessment processes and their outcomes.

**Table 1:** Implementation Matrix for Equity with Van Melle et al.’s 2019 Core Components of CBE.


LEVEL/CORE COMPONENT	1. OUTCOMES & COMPETENCIES DEFINED	2. SEQUENCED PROGRESSION OF COMPETENCE	3. TAILORED LEARNING EXPERIENCES	4. COMPETENCY-FOCUSED INSTRUCTION & ASSESSMENT	5. PROGRAMMATIC ASSESSMENT APPROACH

MACRO (Global, regional, institutional)	National and institutional policy [68] & standards [69]: embedding equity explicitly into accreditation and regulatory frameworks; integrating equity criteria; patient/care partner/community representation and co-creation https://canmedsproject.ca/en/consultations. CBHPE standards and frameworks: co-creation with diverse voices [65]; avoiding assumptions based on contexts, including geography and resource level; collaboratively building contextualized frameworks.	Context-specific (e.g., size of program, level of resources, discipline) recommendations or best practices: for the sequencing of progression Funding & access to opportunities: removing SES-related structural barriers; scholarships, bursaries, and loans for financially unstable learners to provide equitable opportunities during training (core and elective); Pathway programs for individuals with one or more structurally excluded identities starting as early as 4 years old [70,71,72]. We argue that these pathway programs bring needed diversity into the health learner and workforce and potentially set them up for success even before they start, and facilitate smooth progression	Program structure: training opportunities aligned with social accountability mandates; training sites serving structurally excluded communities (including and not limited to patients/care partners from or experiencing rural/remote regions, immigration, refugeeism, long-term care, unhoused, or justice-involved) aka service learning with direct engagement with community and reciprocal partnerships [73,74].	Context-specific (e.g., size of program, level of resources, discipline) recommendations or best practices for equity-informed instruction and assessment [72], which are then adapted to the local environment. Curricular requirements: National program accreditation bodies ensure representation and equity standards as part of their common program requirements and program accreditation standards when reviewing program curricula and evaluation elements	Accreditation of programs: accrediting bodies provide guidelines for equity audits of assessment systems within programs and ensure national accreditation standards. These standards, ranging from certification eligibility to program accreditation, include co-produced elements focused on equity in determining program accreditation and learner progression to unsupervised practice.

MESO (Program/educational institution)	Curricular design: integrating equity across competencies and EPAs [75]; structurally excluded identities as one of many dimensions for case complexity; aligning patient cases with the population served [76].	Admissions: fair access; pathway programs (see above); addressing structural, historic, and current-day barriers.	Institutional support (cross-cutting): the well-being of learners, faculty, and staff; implementation of the Ottawa, Edmonton, Okanagan, and Limerick Charters (https://www.healthpromotingcampuses.org/charters) (well-being, social justice, and planetary health) for health-promoting campuses [77]; inclusive orientation and onboarding [78].	Faculty development: mitigating bias in teaching and assessment [72,79,80]. Assessment practices: equity-informed assessment [72]; coaching mindset; meaningful feedback; development of professional relationships to foster trust and psychological safety in the learning and work environment; structured improvement opportunities; multisource feedback; coaches outside the assessor pool; forward feeding within the CQI culture.	Program evaluation: equity-informed continuous quality improvement (CQI); multisource data; evaluating representativeness of clinical cases/experiences to communities served; mitigating bias at individual, team, and program/institutional levels (through education, ongoing dialogue, equity-informed policy review and creation, etc.) Institutional governance: shared decision-making; learner or patient/care partner/community representation in competence and other curriculum committees.

MICRO (Classroom, clinical, interpersonal)	Coproduction: Outcome competencies are co-created with a diverse representation of learners, faculty, and staff to reflect the local context.	Individualized learning: Learners progress at their own rate, with clear evidence that standards are applied equitably and that there are no differences between groups (broadly defined) in rate of progression, which is audited regularly.	Clinical learning: clinical experiences/rotations in underserved communities; genuine reciprocity with community partners; exposure to diverse contexts reflective of communities served [73,75].	Teacher–learner interaction [72]: inclusive pedagogy; relationship-building, acknowledging lived experience; individualized accommodations. Learning environment: safe, welcoming spaces; addressing microaggressions (racism, gender, disability, neurodiversity, just to name a few) [72]: equity-informed reporting mechanisms; restorative justice approaches to navigating conflict and reporting processes [81,82].	Individual assessment: recognizing diverse ways of demonstrating competence; context-aware WBAs [72,83]. Mentoring & support: mentorship and peer support for underrepresented learners and faculty; tailored support based on individual needs [75,84].


As outlined in the examples above and Table 1, for CBHPE to address rather than reproduce existing inequities, equity must be deliberately embedded at the micro, meso, and macro levels. Kakara Anderson et al. (2025)’s conceptualization of equity through the paradigms of fairness, inclusion, and justice in both design and outcomes is useful in this regard [3]. This framing prompts critical questions: Who defines the competencies? Whose voices are privileged? How is access to additional training, leadership, or scholarship shaped by CBHPE design? However, in much of the current discourse on CBHPE implementation, equity remains implicit, assumed, or treated as peripheral rather than articulated as a core design principle. When not made explicit, equity is unlikely to be operationalized, monitored, or protected throughout implementation and program evaluation. Without intentional multi-level integration, CBHPE risks reinforcing hierarchies, power imbalances, exclusions, and disparities it is intended to address, thereby undermining its social accountability and moral mandate.

Table 1 illustrates how equity can be embedded in each of the five core CBHPE components across levels, providing a structured approach to making equity actionable and consistently integrated across contexts [4]. For example, at the macro level, governments and regulators can include equity and social accountability requirements in defining outcomes and programmatic assessment systems; at the meso level, programs and institutions can tailor learning experiences to social needs and implement equity-informed coaching practices; at the micro level, individualized learning plans can support the sequencing of competencies to mitigate inequities in learner resources.

## Limitations and Future Directions

The English-language literature—and our author team—offered a number of potential solutions across the various domains relevant to the implementation of CBHPE and also highlighted the tensions surrounding its adoption in majority-world contexts. However, there remain substantial opportunities for potential solutions beyond what this group can provide. As an example, the majority of solutions presented in the literature originate from Western settings, and those that do describe majority-world contexts often reflect Western values or import Western CBHPE constructs into non-Western environments [65]. We acknowledge that the limited diversity of the consulted references and our knowledge of locally disseminated case studies may raise questions about the generalizability and scalability of the proposed model across diverse institutional and sociocultural contexts. Unfortunately, the evidence from the literature scan we conducted was sparse. However, the diversity of the author team’s backgrounds and professional and linguistic contexts recognizes this limitation and offers a surrogate lens for understanding it.

Although our author team includes members from both Western and majority-world contexts, we cannot fully capture the breadth of practices and solutions as they are enacted across the diverse majority-world contexts. The publication imbalance between Western and majority-world scholarship, coupled with the unconscious biases inherent to our team, contributes to the proverbial “black box” of understanding what is actually happening on the ground as majority-world institutions continue to adapt, adopt, and implement CBHPE [66].

To close this knowledge gap, we recommend a comprehensive landscape analysis and/or a case compendium documenting how CBHPE is being adopted across majority-world settings. Such work should highlight the infrastructures, contextual adaptations, flexible implementation strategies, and equity-oriented frameworks that enable CBHPE to succeed in widely varied environments. Conducting this work will require significant time, coordinated infrastructure, coalition-building, and sustainable funding—ideally supported in part by better-resourced Western partners—to meaningfully capture the experiences of majority-world stakeholders at the micro, meso, and macro levels.

## Conclusion

This Eye Opener highlights that despite the transformative potential of CBHPE, equity is often not specifically addressed in the CBHPE literature. Nevertheless, the full potential of CBHPE can only be realized across jurisdictions when equity is intentionally embedded at every level of educational design, implementation, and evaluation. At the same time, CBHPE frameworks offer flexibility, transparency, and improved outcomes, global adaptation risks perpetuating existing inequities, especially when education and assessment models are transposed without deliberate and meaningful local adaptation. Local CBHPE progress should be founded on a principled tolerance for contextual variation rather than a fixed view of fidelity. This involves a commitment to co-construction at all levels, with a deliberate focus on fairness, inclusion, and justice. By critically interrogating whose voices are privileged or unheard and by fostering collaborative knowledge exchange between the majority world and high-income countries, and between well-resourced and less-resourced settings, CBHPE can move beyond symbolic inclusion to genuinely disrupt entrenched hierarchies and advance social accountability in health professions education.

Hence, we propose a shift in perspective. From the viewpoint of the Global South, Western nations export CBHPE to the majority world, that is, from the “outside-in”; a counter-model of information sharing is “outside-in, inside-out and all around,” in which CBHPE frameworks are co-created to leverage the contributions of both Western and majority-world education systems. Equity demands that majority-world contexts not merely *receive* CBHPE models, but rather actively *inform and shape* them, enriching global health professions education. Ultimately, thoughtful adaptation and embracing Global South ways of knowing, doing and being, rather than simple replication, are essential to ensure that CBHPE serves all communities and contributes to equitable, high-quality healthcare without discrimination. It means finding that missing E and making equity a constant [67].

## Author reflexivity

The author group comprised a broadly representative, intentionally assembled collective of health profession education scholars with substantive expertise in the design and implementation of CBHPE (in medicine, nursing, and midwifery) across undergraduate, graduate, postgraduate, and faculty development contexts. Scholars with established expertise in social justice within HPE were purposively included to deepen the discourse and strengthen the content expertise informing this work. Members were based in Aruba, the Netherlands, Canada, the United States, the United Kingdom, Spain, Argentina, and Australia, and collectively brought professional experience from multiple global contexts. The group reflected diversity across racial, ethnic, and gender identities, as well as sexual orientation and religious traditions, with many members having immigrated to the countries where they currently live and work. While the majority of the team have experience in the Global North, two team members live, work, learn, and teach in the Global South and in languages other than English.

## Disclosure statement

This article is part of a special series from the International Competency-Based Health Professions Educators Collaborative (ICBHPE). Articles in the special series are work products of an international convening of members of this group from February 10–12, 2025, at the Stanford University School of Medicine (Stanford, California, USA), and of ongoing discussions that followed that in-person forum. These discussions capitalized on broad-based input from the Collaborative. However, the opinions expressed in this article are those of the authors and do not necessarily reflect an official stance or policy of The Collaborative or of the institutions funding the publication of the papers in the special series.
